# Leukocyte Profiles Reflect Geographic Range Limits in a Widespread Neotropical Bat

**DOI:** 10.1093/icb/icz007

**Published:** 2019-04-27

**Authors:** Daniel J Becker, Cecilia Nachtmann, Hernan D Argibay, Germán Botto, Marina Escalera-Zamudio, Jorge E Carrera, Carlos Tello, Erik Winiarski, Alex D Greenwood, Maria L Méndez-Ojeda, Elizabeth Loza-Rubio, Anne Lavergne, Benoit de Thoisy, Gábor Á Czirják, Raina K Plowright, Sonia Altizer, Daniel G Streicker

**Affiliations:** 1 Odum School of Ecology, University of Georgia, Athens, GA 30602, USA; 2 Center for the Ecology of Infectious Disease, University of Georgia, Athens, GA 30602, USA; 3 Department of Biology, Indiana University, Bloomington, IN 47405, USA; 4 Departamento de Ecología, Genética y Evolución, Facultad de Ciencias Exactas y Naturales, Universidad de Buenos Aires, Buenos Aires C1428EGA, Argentina; 5 Department of Microbiology and Immunology, Montana State University, Bozeman, MT 59715, USA; 6 Departamento de Metodos Cuantitativos, Facultad de Medicina, Universidad de la República, Montevideo 11800, Uruguay; 7 Department of Wildlife Diseases, Leibniz Institute for Zoo and Wildlife Research, Berlin 10315, Germany; 8 Department of Zoology, University of Oxford, Oxford OX1 3SY, UK; 9 Facultad de Ciencias, Universidad Nacional de Piura, Piura 20009, Peru; 10 Programa de Conservación de Murciélagos de Perú, Piura Lima-1, Peru; 11 Association for the Conservation and Development of Natural Resources, Lima 15037, Peru; 12 Yunkawasi, Lima 15049, Peru; 13 Departamento de Histología, Facultad de Medicina, Universidad de la República, Montevideo 11800, Uruguay; 14 Department of Veterinary Medicine, Freie Universität Berlin, Berlin 14163, Germany; 15 Facultad de Medicina Veterinaria y Zootecnia, Universidad Veracruzana, Veracruz 91710, Mexico; 16 Centro Nacional de Investigación Disciplinaria en Microbiología Animal, Instituto Nacional de Investigaciones Forestales, Agrícolas y Pecuarias, Mexico City 05110, Mexico; 17 Laboratoire des Interactions Virus-Hôtes, Institut Pasteur de la Guyane, Cayenne, French Guiana F-97300, France; 18 Institute of Biodiversity, Animal Health and Comparative Medicine, College of Medical, Veterinary and Life Sciences, University of Glasgow, Glasgow G12 8QQ, UK; 19 MRC—University of Glasgow Centre for Virus Research, Glasgow G61 1QH, UK

## Abstract

Quantifying how the environment shapes host immune defense is important for understanding which wild populations may be more susceptible or resistant to pathogens. Spatial variation in parasite risk, food and predator abundance, and abiotic conditions can each affect immunity, and these factors can also manifest at both local and biogeographic scales. Yet identifying predictors and the spatial scale of their effects is limited by the rarity of studies that measure immunity across many populations of broadly distributed species. We analyzed leukocyte profiles from 39 wild populations of the common vampire bat (*Desmodus rotundus*) across its wide geographic range throughout the Neotropics. White blood cell differentials varied spatially, with proportions of neutrophils and lymphocytes varying up to six-fold across sites. Leukocyte profiles were spatially autocorrelated at small and very large distances, suggesting that local environment and large-scale biogeographic factors influence cellular immunity. Generalized additive models showed that bat populations closer to the northern and southern limits of the species range had more neutrophils, monocytes, and basophils, but fewer lymphocytes and eosinophils, than bats sampled at the core of their distribution. Habitats with access to more livestock also showed similar patterns in leukocyte profiles, but large-scale patterns were partly confounded by time between capture and sampling across sites. Our findings suggest that populations at the edge of their range experience physiologically limiting conditions that predict higher chronic stress and greater investment in cellular innate immunity. High food abundance in livestock-dense habitats may exacerbate such conditions by increasing bat density or diet homogenization, although future spatially and temporally coordinated field studies with common protocols are needed to limit sampling artifacts. Systematically assessing immune function and response over space will elucidate how environmental conditions influence traits relevant to epidemiology and help predict disease risks with anthropogenic disturbance, land conversion, and climate change.

## Introduction

Environmental changes such as climatic shifts and deforestation alter patterns of infectious disease in wildlife (Acevedo-Whitehouse and Duffus 2009; Brearley et al. 2013). Although environmental variation can affect host–parasite interactions through various mechanisms, changes to host immune defense can have multiple and interacting effects on infection dynamics (Martin et al. 2010; Hawley and Altizer 2011). Environmentally driven differences in immune phenotypes can alter the likelihood of infection given exposure (i.e., susceptibility), clearance of infection, and degree of infection-induced mortality (Jolles et al. 2015). For example, wild bovids in poor nutritional condition are more susceptible to helminth infection (Ezenwa 2004), and variation in inflammatory cytokine signaling between house finch populations likely drives differences in tolerance to *Mycoplasma gallisepticum* (Adelman et al. 2013). Understanding how environmental variation affects host immunity is therefore important for predicting which wild populations may be more vulnerable to emerging pathogens or be involved in pathogen spillover.

Environmental variation in host immune phenotypes can arise through various spatial processes that occur at small and large scales, including parasite risk, abiotic conditions, food availability, and predator abundance (Morand et al. 2010; Albery et al. 2018). For example, at large spatial scales, variation in Lyme disease risk was associated with higher frequency of a protective gene variant of an innate immune receptor in bank vole populations across Europe (Tschirren 2015), and latitudinal gradients in parasite species richness likely have selected for greater host investment in immunity (Møller 1998; Stephens et al. 2016). Small population size at the edges of a host distribution could also shape immunity through recurrent parasite extinctions and reinvasions (Keeling and Grenfell 1997). Proximity to range limits might also compromise immunity due to pronounced physiological stress or energetic requirements in sub-optimal abiotic or biotic conditions, in which animals must allocate more energy into survival and reproduction (Lochmiller and Deerenberg 2000; Romero et al. 2000). For example, tree swallows at the northern extreme of their range display stronger tradeoffs between immunity and reproduction than at the range center (Ardia 2005). Yet at finer spatial scales, local variation in habitat quality could also shape immune phenotypes (Couch et al. 2008; Albery et al. 2018). To identify the spatial scales at which the environment shapes immunity and to differentiate between these various hypothesized covariates, field studies of wild populations must capture environmental variation across habitat gradients; a narrow spatial extent is likely to generate low variation given the principle of spatial autocorrelation (Tobler 1970). However, large-scale sampling efforts across broad geographic distributions are challenging for many wildlife.

Bats are a well suited taxon for quantifying the spatial scale and environmental drivers of immunological variation, because many species are highly mobile and have large geographic ranges that cover multiple habitat types (Jones et al. 2009). Interactions between bats, immunity, and infection are also important for conservation and public health, as bats can be severely affected by fungal diseases such as white-nose syndrome (Frick et al. 2015) and are reservoirs for zoonotic viruses and bacteria (Brook and Dobson 2015). Identifying how environmental conditions shape bat immunity could help explain spatial patterns of parasite diversity and identify colonies likely to be susceptible to new infections or be involved in zoonotic spillover. However, studies of bat immunology have been largely restricted to cell culture and captivity (Zhou et al. 2016) or to a small number of nearby sites (Allen et al. 2009; Schneeberger et al. 2013; Seltmann et al. 2017), limiting large-scale analyses of environmental variation.

We here assess spatial variation in cellular immunity of the common vampire bat (*Desmodus rotundus*) from eight countries spanning the broad distribution of this species across the Neotropics. Vampire bats are habitat generalists that occur in tropical and subtropical regions of Central and South America while also inhabiting more temperate regions as far south as central Chile, Uruguay, and northern Argentina (Greenhall et al. 1983). Vampire bats feed on blood and forage in diverse habitats, including arid coastlines, high-elevation mountains, and lowland rainforests, on prey ranging from tapir and sea lions to cattle and humans (Streicker and Allgeier 2016). Given this feeding behavior, vampire bats can transmit pathogens such as rabies virus, for which this species is the main reservoir in the Neotropics (Schneider et al. 2009). Vampire bats may also transmit other zoonotic pathogens (Tong et al. 2013; Volokhov et al. 2017; Becker et al. 2018a). Understanding the spatial scale at which environmental variation is associated with bat immunity, and the environmental predictors of this variation, could help predict regions of spillover risk, which is of particular concern as climate change facilitates range expansions of both vampire bats and their pathogens (Lee et al. 2012).

Large-scale studies present several logistical issues, including the challenges of synchronizing efforts over space and time, accounting for variable breeding phenology, and keeping methods consistent. While these issues can introduce noise, large-scale studies can still help identify patterns and generate new hypotheses. To present a preliminary spatial analysis of bat immune phenotypes and to efficiently capture variation across a broad spatial extent, we used differential white blood cell (WBC) counts collected from our collective field sites and extracted from the literature to quantify leukocyte profiles (Davis et al. 2008). Neutrophils and monocytes are key components of innate immunity involved in phagocytosis and the inflammatory response against bacteria, protozoa, and fungi (Tizard 2012). Lymphocytes are involved in adaptive immunity (e.g., B and T cells) and signal investment in pathogen-specific defense. Eosinophils and basophils are rarer and can defend against macroparasites (Eberle and Voehringer 2016). While signaling investment in innate versus adaptive cellular immunity, WBC counts can also indicate chronic stress, as the ratio of neutrophils to lymphocytes often scales positively with plasma glucocorticoids, while eosinophil counts can decline (Davis et al. 2008). Leukocyte profiles can correlate with functional immune metrics, such as bacterial killing ability and inflammatory response (Beechler et al. 2012; Zhang and Zhao 2015) and are of diagnostic value in medicine (Walsh et al. 2005). Leukocyte profiles are amenable to comparative studies given the ease of sample collection and small blood volumes (Schneeberger et al. 2013; Maceda-Veiga et al. 2015), allowing us to capitalize on a diverse range of vampire bat research efforts.

We first used multivariate analyses to derive one axis of WBC variation across our bat populations and published summary data. To identify the scale at which leukocyte profiles may be influenced by environmental conditions, we quantified spatial autocorrelation in WBCs. We expected general patterns of autocorrelation declining with distance (Koenig 1999). We then used general additive models (GAMs) to identify environmental predictors of spatial variation using a suite of covariates that captured local biotic, local abiotic, and biogeographical factors. We also assessed the sensitivity of our results to variable time between capture and sampling.

## Methods

### Field data collection

We sampled 572 vampire bats across 33 sites in Peru, Belize, Argentina, French Guiana, Mexico, and Uruguay from 2013 to 2017 (Argibay et al. 2016; de Thoisy et al. 2016; Streicker et al. 2016; Becker et al. 2018b; Escalera-Zamudio et al. 2018; Table 1). Bats were captured in mist nets or harp traps placed at the exists of roosts, along flight paths, outside of livestock corrals, or with hand nets in roosts. Individual-level data on the time between capture and sampling were available for 12 sites (OW1–2, LR1–7, AM1–2, CA1); other sites reported approximate intervals (Supplementary Table S1). Bats were sampled for blood with sterile 23-gauge needles and heparinized capillary tubes at the propatagial vein. Blood smears were stained with Romanowsky stains (Wright–Giemsa or May–Grünwald–Giemsa; Kradin 2017). Most bats were released (bleeding was stopped with styptic gel), but a small fraction were euthanized with ketamine and cervical dislocation. Details of permits and animal use are provided in the Ethics statement. Most data for this study come from one sampling event per site (Table 1); a subset of sites were sampled for blood smears across multiple months, and only five of these sites were sampled for multiple months within a given year (CA1, AM1, AM3, LR2, and LR4).

**Table 1:** Sampling effort for leukocyte profiles of wild *Desmodus rotundus* per country across 39 populations

Country	Sites	*N*	Years
Argentina	1	9	2013
Belize	2	89	2014–16
Brazil	4	69	1997^a^, 1997, 2007^a^, 2010^a^
Costa Rica	1	3	2009, 10
French Guiana	2	8	2017
Mexico	3	31	1939^a^, 2014
Peru	24	420	2013–16
Uruguay	2	18	2017

Presented are the number of sites per country, total sample size (*N*), and sampling years. See Supplementary Table S1 for leukocyte differentials, sample size, median hours from capture to sampling, and spatial coordinates per site or study.

^a^Date of publication, sampling year(s) not reported.

### WBC differentials

We performed differential WBC counts by recording the identity of 100 leukocytes under 1000× magnification with a light microscope (oil immersion) and counting the numbers of neutrophils, lymphocytes, monocytes, eosinophils, and basophils (Houwen 2001). The mean proportion of each WBC, sample size, longitude, and latitude was recorded per site; while medians may be more appropriate given skew in WBC data (Supplementary Fig. S1; Minias et al. 2018), we here used means given that all literature data (see below) reported this summary statistic. WBC data were fairly consistent within each site: coefficients of variation derived from log-transformed data averaged 0.12 for neutrophils, 0.12 for lymphocytes, and under 0.02 for the other leukocytes (Supplementary Fig. S1). We limited our analyses to sites with data from three or more bats (sample size range = 3–60, *x̄* = 17).

We supplemented field data with leukocyte profiles of wild vampire bats identified from a systematic literature search. Searches were run in Google Scholar, Web of Science, CAB Abstracts, and JSTOR using the following term strings: (“*D. rotundus*” OR “vampire bat”) AND (hematology OR leukocyte OR “WBC”). This identified 602 unique studies that we screened for inclusion criteria (differential WBC counts in wild *D. rotundus*), resulting in four studies. We averaged data from two studies in an identical site (Schinnerl et al. 2011; Schneeberger et al. 2013). We included another three studies included in references (Martinez 1939; Baptista and Esbérard 1997; Vilar et al. 2005), producing six total sites from Mexico, Costa Rica, and Brazil (Table 1, Supplementary Fig. S2, and Supplementary Table S1). We extracted the mean proportion of each WBC and sample size. One study did not report sample size (Martinez 1939), which we approximated using mean standard deviation across WBCs and 5% error. We obtained spatial coordinates from each location, as the centroid of a subregion, or by averaging coordinates from multiple sites. No study reported the time between capture and sampling.

### Environmental covariates

We obtained data on local abiotic conditions, local biotic conditions, and biogeographic features of the vampire bat distribution across the species range. We produced a 500 ×500 grid of sampling points across the distribution provided by the IUCN (Barquez et al. 2015). We obtained rasters for abiotic conditions from WorldClim (Hijmans et al. 2005) and used the *raster* package in R to extract data at the resolution of 2.5 min latitude, corresponding to a 5 km radius (Bivand et al. 2008). We extracted local meters altitude (log transformed) and four bioclimatic variables (Supplementary Table S2 and Supplementary Fig. S3): mean annual temperature (°C), temperature seasonality (SD * 100), annual mean precipitation (mm), and precipitation seasonality (SD * 100).

These four local bioclimatic covariates were correlated across the species range: absolute values of correlation coefficients (|*r*|) ranged from 0.06 to 0.62, with a mean of 0.41 (Supplementary Fig. S4). We thus collapsed bioclimatic covariates using a principal component analysis (PCA), with variables centered and scaled to have unit variance. We applied the PCA to the entire grid of bioclimatic covariates so loadings would be equal between the full distributions and sampled sites. We kept the first two environmental principcal components (EPC1 and EPC2) for analyses. EPC1 explained 57% of bioclimatic variation and loaded positively by mean temperature (0.52) and precipitation (0.6) and negatively by temperature seasonality (–0.51) and precipitation seasonality (–0.34); increasing values display increasingly warm and wet habitats with less temperature seasonality (Supplementary Fig. S5). EPC2 explained 24% of bioclimatic variation and loaded positively by precipitation seasonality (0.84) and temperature (0.23) and negatively by temperature seasonality (–0.47) and precipitation (–0.12); increasing values display habitats with more variable rainfall but more constant temperature (Supplementary Fig. S5).

As a proxy for local biotic conditions (e.g., prey abundance), we obtained rasters of the total biomass of mammal livestock (cattle, pigs) and poultry (chickens); we used livestock given the dominance of domestic prey in vampire bat diets and the absence of standardized abundance estimates for wild prey (Bobrowiec et al. 2015). We used the Gridded Livestock of the World database of 2010 modeled livestock abundance estimates and average species mass (kg) from the AnAge Database; we normalized total livestock biomass within a 5-km radius using a quarter-root transformation (Becker et al. 2018b; Gilbert et al. 2018).

**Fig. 1:**
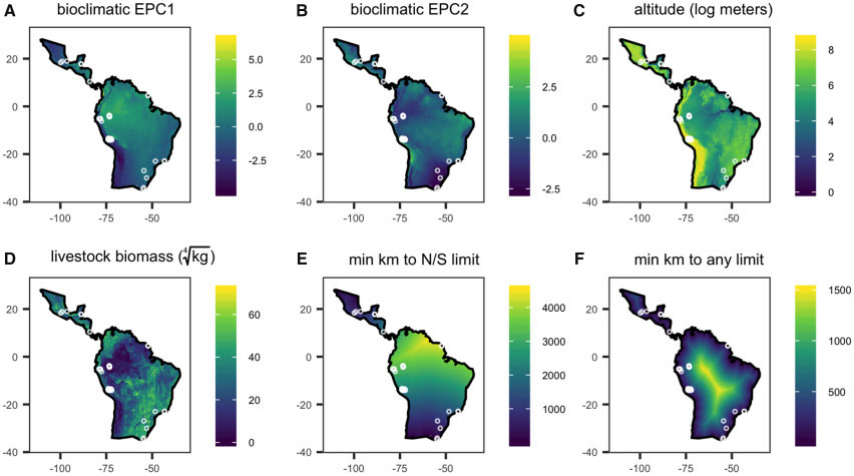
Distribution of local abiotic (**A**–**B**), local biotic (**C**–**D**), and biogeographic covariates (**E**–**F**) across the *Desmodus rotundus* range. Locations of the 39 wild populations are overlaid.

We extracted two biogeographic variables related to the vampire bat distribution. To assess effects of occupying the latitudinal extreme of the species distribution, we measured the minimum distance of each samping location to the northern or southern limit of the range (28°N and 33°S; Greenhall et al. 1983). To differentiate effects of latitudinal extremes from those of being an edge population (e.g., small colonies, extinction–recolonization), we measured the minimum distance of each samping location to any boundary of the bat distribution. Variation in local abiotic conditions (EPC1, EPC2, altitude), local biotic conditions (livestock biomass), and biogeography (distance to the latitudinal limits, distance to any range edge) is shown in Fig. 1.

### Colony-level WBC data

**Fig. 2:**
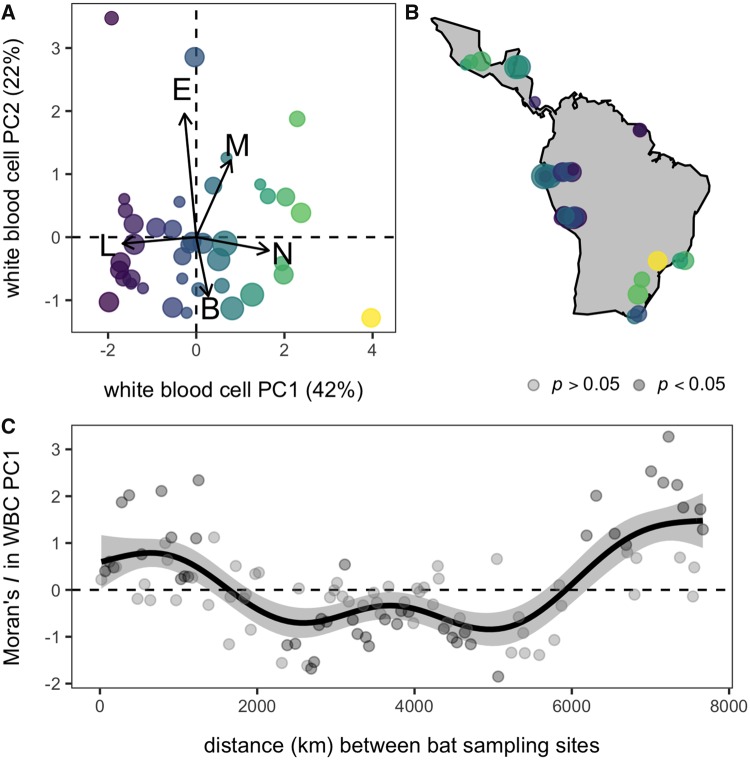
Spatial variation in *Desmodus rotundus* WBC profiles. (**A**) The principal components biplot shows loadings in arrows; site data are scaled by sample size and colored by WBC PC1. (**B**) Corresponding WBC PC1 values are mapped across the species distribution, with points jittered to reduce spatial overlap. (**C**) The spatial correlogram shows the estimated values of Moran’s *I* as a function of distance between sites. Black points show significant negative (*I *<* *0) or positive (*I *>* *0) spatial autocorrelation, with *p-*values generated through 1000 permutations. The black line and gray band show the fitted mean and 95% confidence interval using a GAM.

As WBCs function interdependently and as proportions of each cell represent compositional data, we performed a PCA to reduce the biologically and statistically dependent proportions of each cell into a single axis using our site-level means (Matson et al. 2006; Buehler et al. 2011). The first PC (WBC PC1) explained 42% of cellular variation and was the only axis supported by Horn’s parallel analysis (λ = 1.66). WBC PC1 loaded positively by neutrophils (0.66), monocytes (0.31), and basophils (0.11) and negatively by lymphocytes (–0.67) and eosinophils (–0.11; Fig. 2A). We did not separately analyze the ratio of neutrophil to lymphocyte given the high loadings of these cells, the strong correlation with WBC PC1 (*r *=* *0.9), and as integrating information on other cells can more holistically inform stress and cellular immunity (Davis et al. 2008).

### Statistical analyses

All analyses were performed in R (R Core Team 2013). We estimated spatial autocorrelation in WBC PC1 with Moran’s *I* using the *ape* package to test whether closely sampled bat populations share more similar leukocyte profiles (Paradis et al. 2004). We estimated the correlogram at 50 km intervals using the *ncf* package to identify the scales at which autocorrelation occurs (Bjørnstad and Falck 2001). We then used a GAM with a penalized thin plate spline using the *mgcv* package to derive the mean Moran’s *I* as a function of distance (Wood 2006).

We next used GAMs to assess the relationships between environmental covariates and bat leukocyte profiles. We weighted observations by the square-root of sample size to account for variable sampling effort (Garamszegi 2014). To assess the relative support for local abiotic, local biotic, and biogeographic predictors, we built an *a priori* set GAMs using our six environmental covariates (EPC1, EPC2, altitude, livestock biomass, distance to the latitudinal limits, and distance to any range edge). All models used penalized thin plate splines and included the interaction between longitude and latitude to control for spatial dependence (Wood 2006). To restrict the number of possible models, we excluded other interactions and limited GAMs to a maximum of three covariates (e.g., two fixed effects and spatial coordinates); we excluded EPC1 and distance to latitudinal limits from the same GAM due to high collinearity (*r *=* *0.72, Supplementary Fig. S5). We used the *MuMIn* package and Akaike information criterion corrected (AICc) for small sample size to derive relative importance per covariate (Bartoń 2013). We considered models within two ΔAICc to be competitive, using model averaging when appropriate. Lastly, we used the most competitive model(s) to generate a prediction map of leukocyte profiles across the bat range.

### Sensitivity to handling time

Unlike plasma corticosterone, which can increase within minutes of acute stress from capture (Romero and Reed 2005), leukocyte profiles shift more slowly in response to stressors and can remain at baseline during handling (Davis et al. 2008). While bat immune systems may be more robust to acute stress than those of other taxa (Becker et al. 2019), we assessed sensitivity of our results to variable handling time with our field data. Where individual data on handling time were available (12 sites, *n *=* *205), we used a permutational multivariate analysis of variance (PERMANOVA) on the proportions of each leukocyte and a linear model with WBC PC1 as the response; both included the interaction between handling time and site.

We classified median handling time per site as under 1 h (*n *=* *7), under 2 h (*n *=* *6), under 5 h (*n *=* *17), and over 5 h (*n *=* *3; AR1, MX1–2) and included this in a linear model. We used *post hoc* comparisons to identify the period where handling stress was associated with WBC PC1. We then refit the spatial correlogram and top GAM(s) excluding site data where handling time exceeded this threshold as well as literature data (which did not report time).

## Results

### Spatial autocorrelation in leukocyte profiles

Leukocyte profiles varied markedly across the 39 vampire bat populations (Fig. 2A–B); site-level proportions of neutrophils ranged from 0.21 to 0.91, lymphocytes from 0.11 to 0.68, monocytes from 0.00 to 0.09, eosinophils from 0.00 to 0.04, and basophils from 0.00 to 0.03. WBC PC1 showed significant global spatial autocorrelation (Moran’s *I *=* *0.32, *P *<* *0.01), and the GAM showed that local Moran’s *I* varied with inter-site distance (*F*_5.4,__6_ =19.04, *P *<* *0.001). We found significant positive autocorrelation at distances under 2000 km, significant negative autocorrelation at moderate distances of 2000–6000 km, and significant positive autocorrelation at large distances (>6000 km). Leukocyte profiles were therefore most similar to one another at both local and very broad spatial scales; however, estimates of Moran’s *I* in WBC profiles were notably higher at larger spatial scales, consistent with the existence of broad biogeographic drivers (Fig. 2C).

### Environmental predictors of WBC variation

**Fig. 3:**
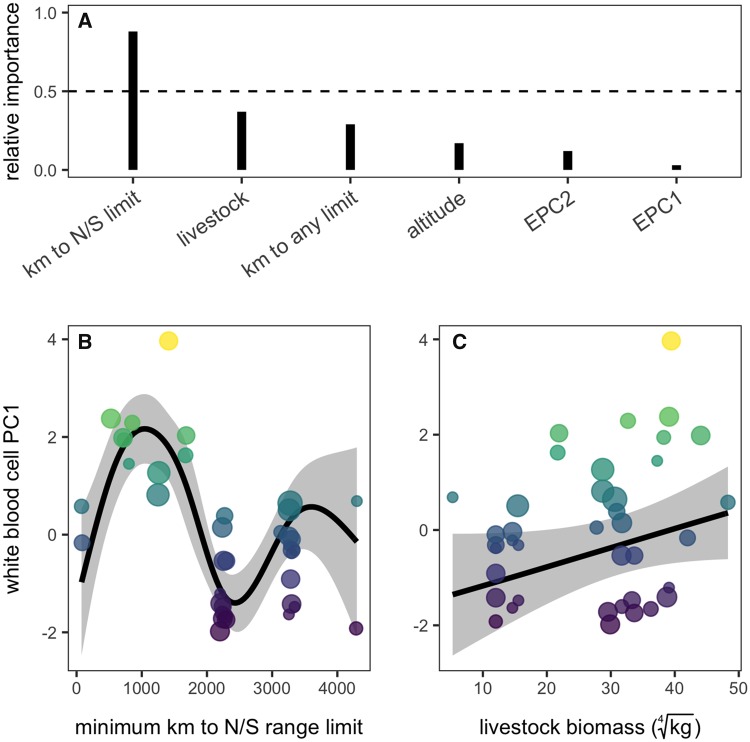
Comparison of local abiotic, local biotic, and biogeographic predictors of *D. rotundus* leukocyte profiles. (**A**) The relative importance of each predictor from the GAM comparison; spatial structure is excluded as it was present in all models. (**B**–**C**) Black lines and gray bands show the fitted means and 95% confidence intervals from the most competitive GAM; data are scaled by sample size and colored by WBC PC1.

Following the spatial correlogram analysis, distance from the northern or southern range limit was the most important predictor of vampire bat leukocyte profiles (relative importance of 88%); local biotic conditions (i.e., livestock biomass) had secondary importance (37%), while distance from any range limit, altitude, and bioclimatic axes (EPC1, EPC2) was relatively uninformative (<30%; Fig. 3A). Accordingly, a GAM with smoothed terms for distance to the latitudinal limits and livestock biomass was the most competitive according to AICc (*w_i_ *=* *0.34; Table 2). WBC PC1 showed a nonlinear and stepwise association with distance (*F*_4.8,__6_ =11.76, *P *<* *0.001); with the exception of two Uruguay sites, WBC PC1 was maximized close to the northern and south range limits and sharply declined for sites sampled 1000–2000 km from range limits (Fig. 3B). WBC PC1 was also weakly linearly related to livestock biomass (*F*_0.8,__6_ =0.57, *P *=* *0.04); sites with more livetock had more neutrophils, monocytes, and basophils and fewer lymphocytes and eosinophils (Fig. 3C). GAMs that included distance from the northern or southern range limit as well as either distance from any range limit or altitude were also competitive (Table 2); however, both predictors had generally weaker effects (distance from any range limit: *F*_0.8,__6_ =0.55, *P *=* *0.04; altitude: *F*_0.6,__6_ =0.24, *P *=* *0.10; Supplementary Fig. S7) and had lower relative importance scores (Fig. 3C). We found no evidence of spatial autocorrelation in any of the GAM residuals (Table 2 and Supplementary Fig. S8).

**Table 2:** Candidate GAMs predicting *Desmodus rotundus* leukocyte profiles

GAM structure	ΔAICc	*w_i_*	*R* ^2^	*I*
∼s(km to N/S limit)+s(livestock)+s(longitude, latitude)	0	0.34	0.71	−0.15
∼s(km to any limit)+s(km to N/S limit)+s(longitude, latitude)	0.6	0.25	0.71	−0.18
∼s(km to N/S limit)+s(altitude)+s(longitude, latitude)	1.99	0.12	0.69	−0.15
∼s(km to N/S limit)+s(longitude, latitude)	2.74	0.09	0.67	−0.09
∼s(km to N/S limit)+s(EPC2)+s(longitude, latitude)	2.75	0.08	0.67	−0.09
∼s(EPC1)+s(altitude)+s(longitude, latitude)	6.37	0.01	0.63	−0.06
∼s(km to any limit)+s(EPC2)+s(longitude, latitude)	7.41	0.01	0.61	−0.09
∼s(km to any limit)+s(longitude, latitude)	7.41	0.01	0.61	−0.09
∼s(km to any limit)+s(livestock)+s(longitude, latitude)	7.41	0.01	0.61	−0.09
∼s(EPC2)+s(longitude, latitude)	7.41	0.01	0.61	−0.09
∼s(longitude, latitude)	7.41	0.01	0.61	−0.09
∼s(km to any limit)+s(altitude)+s(longitude, latitude)	7.42	0.01	0.61	−0.09
∼s(EPC2)+s(livestock)+s(longitude, latitude)	7.42	0.01	0.61	−0.09
∼s(altitude)+s(longitude, latitude)	7.43	0.01	0.61	−0.09
∼s(EPC2)+s(altitude)+s(longitude, latitude)	7.43	0.01	0.61	−0.09
∼s(livestock)+s(longitude, latitude)	7.43	0.01	0.61	−0.09
∼s(altitude)+s(livestock)+s(longitude, latitude)	7.44	0.01	0.61	−0.09
∼s(EPC1)+s(longitude, latitude)	9.13	0	0.54	−0.07
∼s(EPC1)+s(EPC2)+s(longitude, latitude)	9.13	0	0.62	−0.1
∼s(km to any limit)+s(EPC1)+s(longitude, latitude)	9.13	0	0.62	−0.1
∼s(EPC1)+s(livestock)+s(longitude, latitude)	11.71	0	0.55	−0.06
∼1	34.74	0	0	0.34

Competing models are ranked by ΔAICc with Akaike weights (*w_i_*), adjusted *R*^2^, and Moran’s *I* for GAM residuals.

**Fig. 4:**
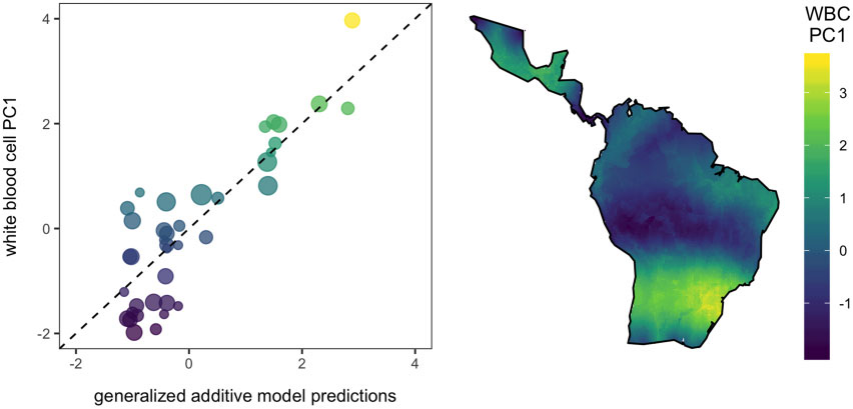
Predictions from the GAM are plotted against observed values and the one-to-one line, with points scaled by sample size and colored by WBC PC1. Corresponding spatial predictions of leukocyte profiles (WBC PC1) are displayed across the *D. rotundus* distribution.

The three top models had strong explanatory power (*R*^2^* *=* *69–71%; Fig. 4). Applying the model-averaged GAM coefficients across the species distribution predicted that vampire bat populations from northern Mexico, Yucatán Peninsula, southern Brazil, northern Argentina, Paraguay, and Uruguay will have greater proportions of neutrophils and monocytes and fewer proportions of lymphocytes and eosinophils (Fig. 4). While these regions mostly reflect the northern and southern limit of the species distribution, WBC PC1 values at localized regions at the center of the species distribution, such as the eastern Brazilian Amazon, Colombia, Ecuador, Venezuela are secondarily driven by livestock biomass and to a lesser extent altitude. Together with the spatial correlogram and variable importance scores, these results highlight that geographic range limits and local biotic conditions play a stronger role in predicting vampire bat leukocyte profiles than local abiotic conditions *per se* and coastal borders of the distribution.

### Sensitivity to handling time

Results were generally robust to variation in handling time. When assessing individual-level relationships, we found no effect of handling time on a per-site basis in the PERMANOVA (*R*^2^* *=* *0.03, *P *=* *0.72) or the linear model (*F*_7,__1__6__7_ =1.05, *P *=* *0.41). Mean handling time per site for field data was strongly associated with WBC PC1 (*F*_3,__2__9_ = 5.41, *P *=* *0.004); however, a *post-hoc* analysis showed that this was driven only by sites where bats were held for over 5* *h (Supplementary Fig. S9). We thus excluded these three sites and literature data for more conservative tests. For the remaining 30 sites, we still observed significant variation in Moran’s *I* as a function of distance (*F*_2.5,__6_ =2.43, *P *<* *0.01), but the effect size was weaker than the original analysis, and most autocorrelation was observed at large distances (Supplementary Fig. S10). Refitting the top GAM still found a nonlinear effect of distance to range limits (*F*_2,__6_ =1.77, *P *<* *0.01) but no effect of livestock biomass (*F*_0,__6_ =0, *p *=* *0.58); the effect of distance was also a saturating decline in WBC PC1 rather than the original stepwise pattern (Supplementary Fig. S10). Spatial predictions again highlighted northern Mexico and the very southern region of the species distribution as having high WBC PC1 (Supplementary Fig. S10), but patterns were not as pronounced as with the full dataset. This discrepancy likely stems from removing WBC data from Brazil, Argentina, and Mexico sites, of which two-thirds were from the literature and thus did not report handling time. When we repeated this sensitivity analysis but retained literature data (*n *=* *36 sites), patterns were more consistent with results of the primary analyses (Supplementary Fig. S11).

## Discussion

Quantifying how immune phenotypes vary spatially, and differentiating between environmental predictors of this variation, is important to ultimately understand landscape-scale differences in host defense. Our spatial analysis of leukocyte profiles in a widely distributed bat species shows that WBCs were autocorrelated at small and large scales, suggesting that not only local environmental conditions but also conditions shared by the extremes of the host distribution predict cellular aspects of the immune system. Comparison among environmental covariates suggested that the latitudinal range limits and local livestock biomass were stronger predictors of WBC variation than local abiotic conditions or coastal range margins. This suggests that colonies close to the latitudinal limits of the species distribution and in habitats with more livestock prey have leukocyte profiles characterized by innate immune cells, including neutrophils and monocytes, relative to lymphocytes and eosinophils. Given our broad spatial sampling extent across the vampire bat distribution, our study provides preliminary but novel insights into how immune phenotypes are associated with spatial scale and environmental variation.

Our finding that bats at the northern and southern range limits generally had relatively more neutrophils, fewer lymphocytes, and fewer eosinophils suggests that the lower quality or suitability of these habitats could cause chronic stress or that bats occupying range limits are in poorer physiological condition (Davis et al. 2008; Morand et al. 2010). In addition to the positive loadings of neutrophils and negative loadings of lymphocytes, WBC PC1 was loaded positively (yet weakly) by basophils, which can increase from chronic stress (Maxwell et al. 1992). Similarly, a small-scale study of great tits found that an edge population had more heterophils (the avian equivalent to neutrophils) and fewer lymphocytes than a core population (Krama et al. 2013), and this is consistent with baseline corticosterone increasing with latitude (Bókony et al. 2009). This association reflects Schmalhausen’s Law, which predicts that animals at the boundary of their tolerance are more frequently exposed to environmental variation (Lewontin and Levins 2000). While we found little signal of local bioclimatic variation directly shaping WBCs (EPC1 or EPC2; Fig. 3), our main axis of bioclimatic variation was partly collinear with the latitudinal range limits (Supplementary Fig. S5), suggesting that these marginal habitats are generally cooler, drier, and exhibit more seasonality in rainfall and temperature. These more variable environments are physiologically relevant, as vampire bats are highly sensitive to cold and dehydration due to their protein-based diet, inadequate lipid stores, and rapid evaporative water loss (Breidenstein 1982). Cooler, drier, and more variable climates increase the energy bats must expend to maintain normal temperature, and thus bats in temperate regions of their range require bloodmeals twice as large as bats in the tropics (McNab 1973). As bloodmeal size is limited by body size and flight capacity, such abiotic conditions may impose stressful conditions; indeed, northern and southern limits of the vampire bat distribution are correlated with the 10°C minimal winter isotherm (McNab 1973). Climate-driven physiological limitations for marginal populations could elevate chronic stress and modulate bat immune phenotypes (Martin 2009).

An alternative explanation for the observed spatial pattern in leukocyte profiles could involve parasite pressure. Work on fruit flies suggests parasite risk to be a stronger predictor of susceptibility than temperature and precipitation (Corby-Harris and Promislow 2008), and higher proportions of neutrophils and monocytes can also indicate inflammation in response to infection (Davis et al. 2008). However, previous work on latitudinal gradients in parasite richness (often being greatest near the equator) casts doubt on parasite pressure driving the observed leukocyte patterns, as neutrophilia would be predicted to instead be greater at the range center (Morand et al. 2010; Stephens et al. 2016). Alternatively, the proximity of the bat range center to the equator could explain the greater investment in adaptive immunity (i.e., lymphocytes) in bat populations farther from the latitudinal range limit, as sympatry is greatest in the tropics (Stevens 1989) and can facilitate inter-species transmission of viruses in bats (Streicker et al. 2010).

In addition to identifying a strong influence of latitudinal range limits on bat leukocyte profiles, we also found that local food abundance (i.e., livestock prey) weakly predicted WBC profiles. This finding builds upon more regional work showing that bats in livestock-dense environments had immune phenotypes skewed toward innate rather than adaptive components and that this pattern was driven more by habitat than individual diet (Becker et al. 2018b). More abundant prey could stimulate bat demography in ways that increase colony size, producing negative feedbacks with chronic stress through resource overmatching and high bat population densities (Murray et al. 2016). Our spatial predictions further suggest that effects of livestock intensification on immunity may be most pronounced at vampire bat range limits.

As a caveat, we had limited ability to control for time between capture and sampling, which can influence leukocyte profiles (Davis et al. 2008); while our main result on latitudinal limits was robust in more conservative analyses, handling time partly obscured large-scale relationships with livestock biomass. We thus highlight the need for spatially and temporally coordinated field studies that use standard protocols across sites to minimize effects of such sampling artifacts. Such work could also overcome the limitations of aggregating field data and published literature, such that studies could include both site-level predictors of immunological variation alongside individual-level (e.g., age, sex, reproduction) and temporal covariates (e.g., monthly and annual variation) while controlling for pseudoreplication and spatial dependence with site random effects (Becker et al. 2018b; Phelps and Kingston 2018).

While use of leukocyte profiles limits inference about functional immunological traits such as susceptibility, tolerance, or recovery (Jolles et al. 2015), predictable spatial variation in WBCs hints that such traits could also vary spatially in ways that alter pathogen transmission. For example, environmentally driven variation in lymphocyte counts, if manifested in weaker cell-mediated defenses (e.g., cytotoxic T cells), could set the stage for uneven responses of bat antiviral defenses to anthropogenic disturbances and generate areas of high spillover risk. Future work could test this prediction by (i) quantifying bat antiviral function and (ii) comparing pathogen diversity or prevalence across the bat range to this predicted leukocyte landscape.

Beyond vampire bats, our study shows that local biotic factors and biogeography can predict immune phenotypes across populations of a broadly distributed species. We capitalized on and aggregated data from disparate sampling efforts and the literature, although future applications of this landscape immunology perspective with standardized field protocols over space and time could provide more robust findings on spatial patterns in leukocyte profiles and if these are moderated by temporal (e.g., seasonality) and individual covariates (e.g., reproduction). Further studies across other widespread species would also benefit from quantifying not only leukocyte profiles but also functional defense (e.g., microbicidal assays), immune genes (e.g., for cytokines, major histocompatibility complex, toll-like receptors), and comprehensive RNA-Seq of immunological organs such as spleen (Fassbinder-Orth 2014). To disentangle the contribution of parasite pressure from local environment and biogeography, future work will also need to quantify not only immune phenotypes but also parasite diversity. This could target pathogens expected to exert selective pressure and use less-biased methods such as metagenomic sequencing of samples pooled per site (Bergner et al. 2018). Applying these tools to robust sampling regimes of widespread host species will provide novel insights into the macroecological drivers of immune defense and spatial variation in host susceptibility.

## Data accessibility

Site-level proportions of each leukocyte type, sample size, median handling time, and spatial coordinates are provided in Supplementary Table S1.

## Ethics

Field methods were approved by the Animal Care and Use Committees of UGA (AUP A2009-10003-0 and A2014 04-016-Y3-A5), Montana State University (2017-30), Universidad de la República (481-2017), and Leibniz Institute for Zoo and Wildlife Research (2012-09-05). Bat capture and sampling were authorized by the Belize Forest Department under permits CD/60/3/14(27), CD/60/3/15(21), and WL/1/1/16(17); by the Peruvian Government under permits RD-273-2012-SERFOR-DGGSPFFS, RD-009-2015-SERFOR-DGGSPFFS, RD-264-2015-SERFOR-DGGSPFFS, and RD-142-2015-SERFOR-DGGSPFFS; by the Uruguayan Government under permit Res. DF137/16; by the Misiones Province Ministry of Ecology and Natural Resources under permit No. 027/2014; and by Mexican regulations under permits Num/SGPA/DGVS 03173/14 and SAGARPA 241111524599811488A467371.

## Supplementary Material

icz007_Supplementary_Data
