# Supplementary material for: Leukocyte Profiles Reflect Geographic Range Limits in a Widespread Neotropical Bat
Source: Integr Comp Biol. 2019 Apr 27;59(5):1176–89. doi: 10.1093/icb/icz007 (PMC6907035; doi:10.1093/icb/icz007)

Leukocyte profiles reflect geographic range limits in a widespread Neotropical bat: Supplemental Information

S1. Field data consistency
S2. Literature search
S3. Site-level leukocyte data
S4. Bioclimatic PCA
S5. Collinearity in predictors
S6. GAM results
S7. Holding time relationships

S1. Field data consistency

Figure S1. Consistency of white blood cell data within the 33 sampled field sites. Left panels show the distribution of each leukocyte type per site, while right panels show the coefficient of variation (CV) for log-transformed data due to the observed skew for most cells and sites. Sites are colored and sorted by geographic region (country or regional identification codes).

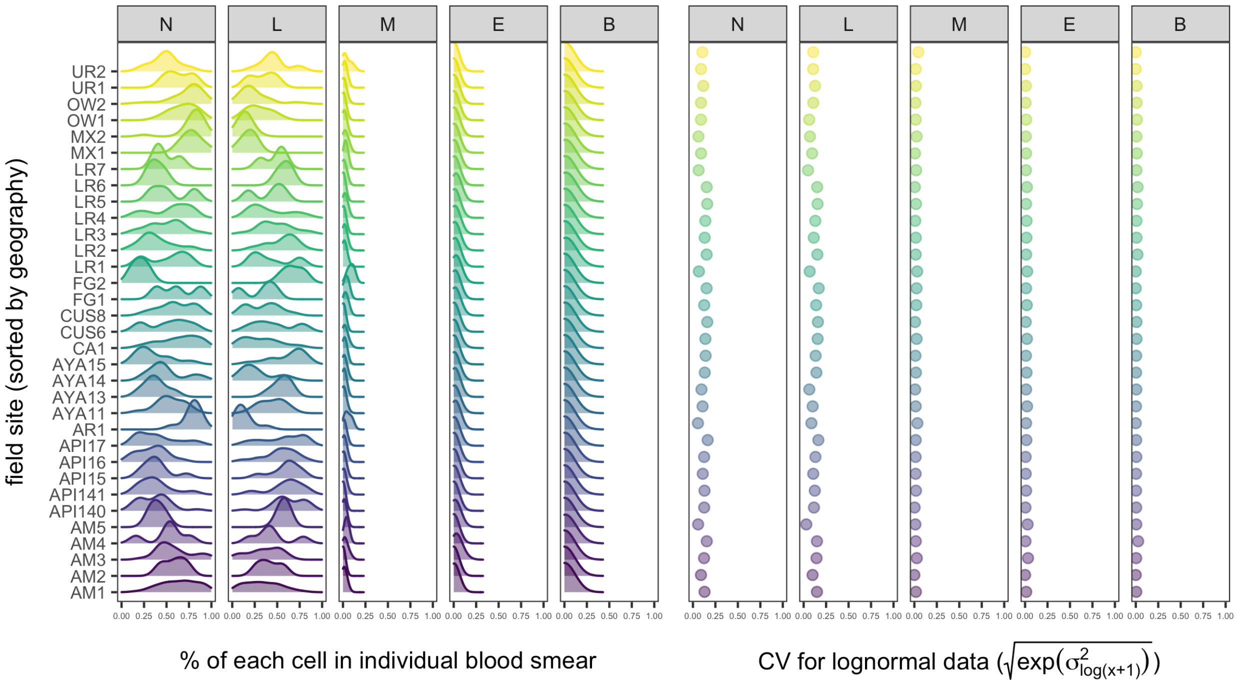

S2. Literature search

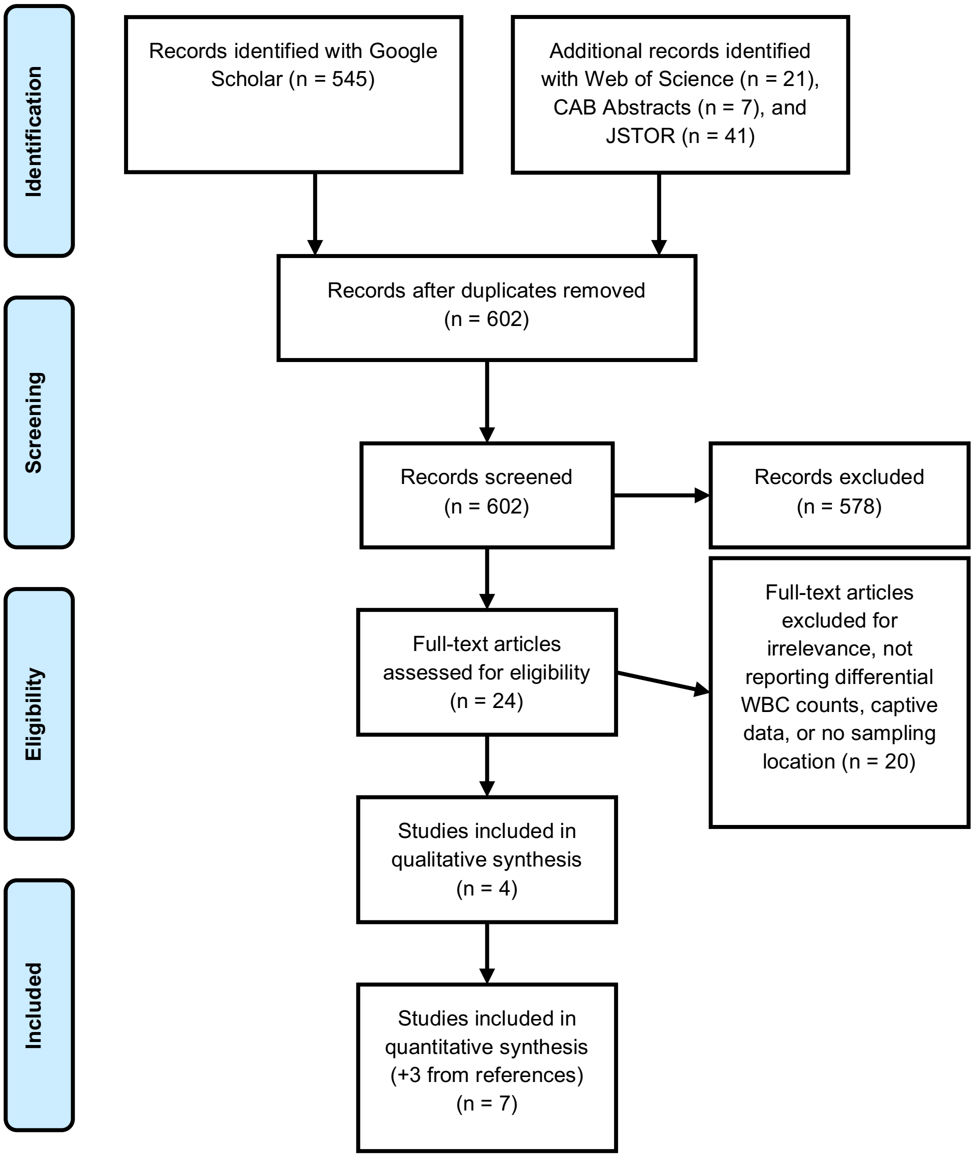

Figure S2. PRIMSA diagram documenting the data collection and inclusion process.

S3. Site-level white blood cell data

Table S1. Proportion of white blood cells from 39 vampire bat populations (site averages), site, code, sample size, country (CR=Costa Rica, FG=French Guiana; AR=Argentina; UR=Uruguay), spatial coordinates, sampling years, and median time between capture and sampling (hours). Numbers are rounded to two decimal places to facilitate display.

Site	%N	%L	%M	%E	%B	*N*	Country	Longitude	Latitude	Years	Hours
AR1	0.79	0.13	0.06	0.01	0.00	9	AR	-54.52	-26.92	2013	over 5
OW1	0.68	0.30	0.01	0.00	0.01	44	Belize	-88.73	17.82	2014–16	< 5
OW2	0.73	0.25	0.01	0.00	0.01	45	Belize	-88.65	17.75	2014–16	< 5
[1]	0.77	0.15	0.04	0.01	0.00	17	Brazil	-43.20	-22.90	1997*	NR
[2]	0.72	0.20	0.04	0.01	0.00	8	Brazil	-43.38	-22.95	1997	NR
[3]	0.85	0.11	0.03	0.01	0.00	24	Brazil	-53.00	-30	2007*	NR
[4]	0.91	0.11	0.08	0.00	0.03	20	Brazil	-48.27	-22.98	2010*	NR
[5,6]	0.42	0.55	0.01	0.01	0.02	3	CR	-84.02	10.42	2009, 10	NR
FG1	0.63	0.30	0.04	0.02	0.00	3	FG	-52.27	4.65	2017	< 1
FG2	0.201	0.68	0.09	0.03	0.00	5	FG	-52.15	4.53	2017	< 1
[7]	0.54	0.27	0.09	0.00	0.00	3	Mexico	-99.88	18.31	1939*	NR
MX1	0.78	0.18	0.03	0.01	0.01	6	Mexico	-99.10	18.87	2014	over 5
MX2	0.80	0.14	0.01	0.01	0.01	22	Mexico	-96.46	18.99	2014	over 5
AM1†	0.64	0.33	0.02	0.01	0.00	41	Peru	-78.29	-5.21	2013, 15–16	< 1
AM2	0.59	0.41	0.00	0.00	0.00	3	Peru	-78.29	-5.21	2015	< 1
AM3†	0.58	0.35	0.03	0.04	0.00	23	Peru	-78.29	-5.20	2013	< 5
AM4	0.51	0.44	0.04	0.00	0.01	5	Peru	-77.64	-6.20	2013	< 5
AM5	0.39	0.57	0.01	0.02	0.00	3	Peru	-78.27	-5.21	2013	< 5
API140	0.37	0.63	0.00	0.00	0.00	24	Peru	-73.14	-14.14	2015	< 5
API141	0.38	0.60	0.01	0.01	0.00	23	Peru	-72.86	-13.79	2015	< 5
API15	0.39	0.60	0.01	0.00	0.00	8	Peru	-73.81	-13.49	2015	< 5
API16	0.34	0.53	0.01	0.01	0.01	15	Peru	-72.36	-13.76	2015	< 5
API17	0.41	0.55	0.01	0.01	0.00	28	Peru	-72.06	-14.08	2015	< 5
AYA11	0.54	0.44	0.01	0.01	0.00	16	Peru	-73.84	-13.42	2015	< 5
AYA13	0.38	0.55	0.01	0.01	0.00	6	Peru	-73.90	-13.95	2015	< 5
AYA14	0.50	0.28	0.02	0.01	0.00	27	Peru	-73.91	-13.93	2015	< 5
AYA15	0.36	0.62	0.02	0.00	0.00	17	Peru	-73.87	-13.47	2015	< 5
CA1†	0.64	0.33	0.02	0.01	0.00	60	Peru	-78.95	-5.17	2013, 15–16	< 1
CUS6	0.52	0.46	0.01	0.00	0.01	25	Peru	-72.25	-13.65	2015	< 5
CUS8	0.61	0.34	0.03	0.02	0.00	13	Peru	-72.51	-13.49	2015	< 5
LR1	0.51	0.44	0.02	0.02	0.02	14	Peru	-73.30	-4.24	2014–16	< 2
LR2†	0.39	0.56	0.02	0.01	0.01	22	Peru	-73.20	-4.29	2014–16	< 1
LR3	0.49	0.48	0.01	0.01	0.00	21	Peru	-73.22	-4.31	2014–16	< 2
LR4†	0.54	0.41	0.02	0.01	0.01	17	Peru	-73.20	-4.21	2014–16	< 2
LR5	0.55	0.41	0.00	0.03	0.02	3	Peru	-73.30	-4.22	2014	< 1
LR6	0.39	0.58	0.01	0.01	0.01	3	Peru	-73.37	-3.77	2014	< 2
LR7	0.49	0.47	0.03	0.01	0.01	3	Peru	-73.36	-3.83	2014	< 5
UR1	0.64	0.34	0.02	0.00	0.00	7	UR	-54.99	-34.63	2017	< 2
UR2	0.49	0.47	0.05	0.00	0.00	11	UR	-54.61	-34.08	2017	< 2

*Date of publication, sampling year(s) not reported.

†Sites with longitudinal leukocyte profile data.

References for literature data

1. Baptista M, Esbérard CEL. 1997 Valores hematológicos de Artibeus sp. e Desmodus rotundus (Mammalia, Chiroptera). *Revista Cientifica do Instituto de Pesquisas Gonzaga Gama Filho* **3**, 11–22.

2. Vilar T, Volino Vaz F, Gitti C, Cordeiro A, Lobo R, Fonseca U, Almosny N. 2005 Hematologic values of Desmodus rotundus (Geoffroy, 1890) in Rio de Janeiro. *Rev Univ Rural* **25**, 57–58.

3. dos Santos AP, Mottin VD, Aita RS, Franciscatto C, dos Anjos Lopes ST, dos Santos Franco W, Hermann GP. 2007 Valores hematológicos e bioquímicos de morcegos hematófagos (Desmodus rotundus rotundus) no sul do Brasil. *Acta Scientiae Veterinariae* **35**, 55–58.

4. Almeida BF, Barbosa TS, Ciarlini LDRP, Pedro WA, Beluccio ML, Queiroz LH, Ciarlini PC. 2010 Valores hematológicos de morcegos hematófagos Desmodus rotundus (E. Geoffroy, 1810) mantidos em cativeiro. *Chiroptera Neotropical* **16**, 780–785.

5. Schneeberger K, Czirják GÁ, Voigt CC. 2013 Measures of the Constitutive Immune System Are Linked to Diet and Roosting Habits of Neotropical Bats. *PLoS ONE* **8**, e54023. (doi:10.1371/journal.pone.0054023)

6. Schinnerl M, Aydinonat D, Schwarzenberger F, Voigt CC. 2011 Hematological survey of common neotropical bat species from Costa Rica. *Journal of Zoo and Wildlife Medicine* **42**, 382–391.

7. Martinez L. 1939 Segunda contribución acerca de la hematometria de los murciélagos mexicanos. In *Anales Instituto de Biología Universidad Nacional Autónoma de México*, pp. 109–113.

S4. Bioclimatic PCA

Table S2. Bioclimatic variables and definitions from WorldClim.

WorldClim Code	Variable
BIO1	Annual Mean Temperature
BIO4	Temperature Seasonality (standard deviation *100)
BIO12	Annual Precipitation
BIO15	Precipitation Seasonality (Coefficient of Variation)

Figure S3. Distribution of the four WorldClim abiotic covariates across the vampire bat geographic range. Locations of the 39 sampled wild populations are provided as points.

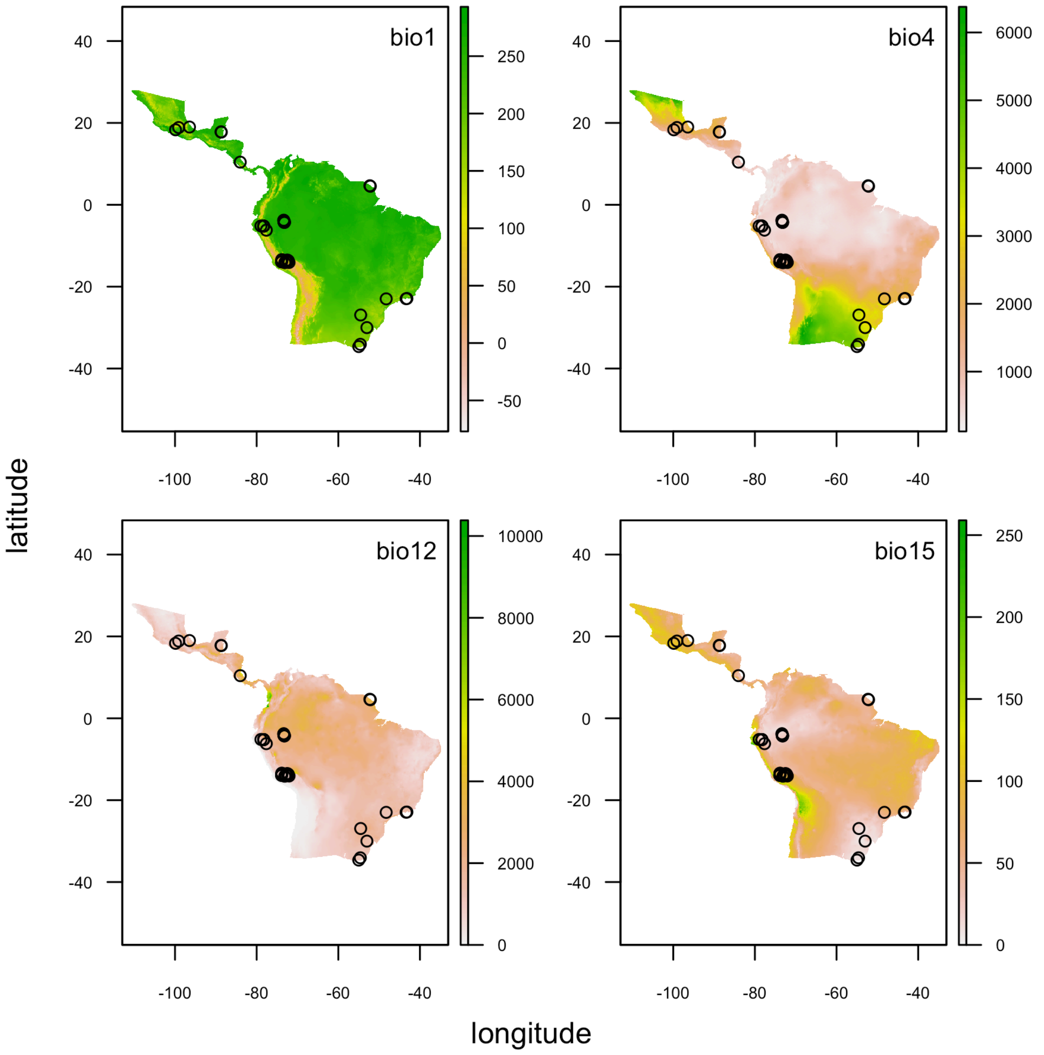

Figure S4. Correlation matrix of WorldClim bioclimatic variables (shown are 250 randomly sampled points from the vampire bat geographic range).

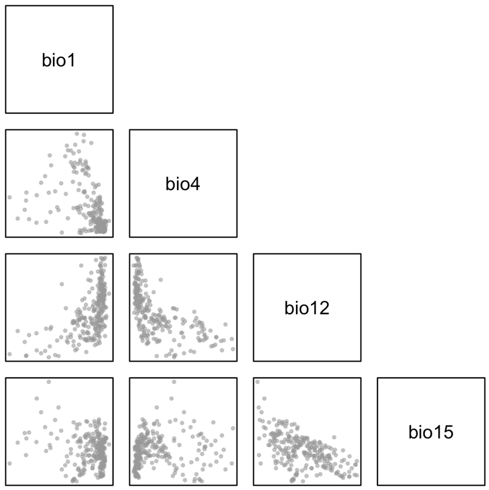

Figure S5. Biplots for the first two bioclimatic EPCs, with loadings displayed with arrows. 1000 randomly sampled points from the vampire bat range are shown in grey, and extracted values for our 39 wild populations are shown in black.

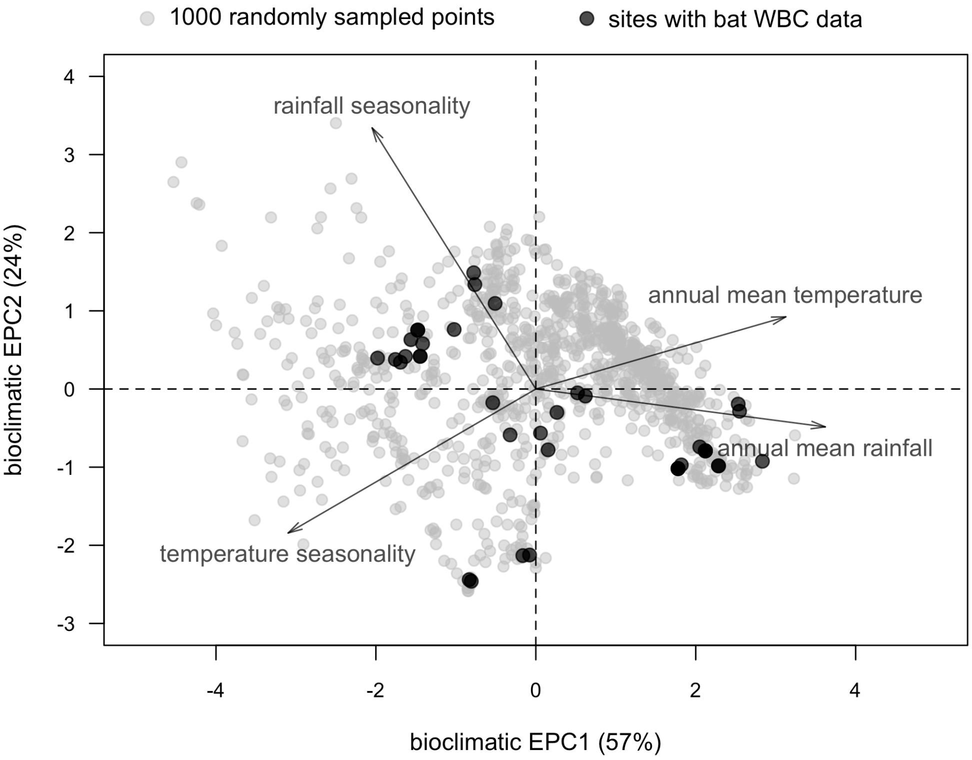

S5. Collinearity in predictors

Figure S6. Correlation matrix of local abiotic (EPC1, EPC2, altitude), local biotic (livestock), and biogeographic predictors (distance to latitudinal limits, distance to any edge). Shown are 1000 points randomly sampled from the vampire bat distribution. Only EPC1 and distance to latitudinal limits showed substantial collinearity (*r*=0.71).

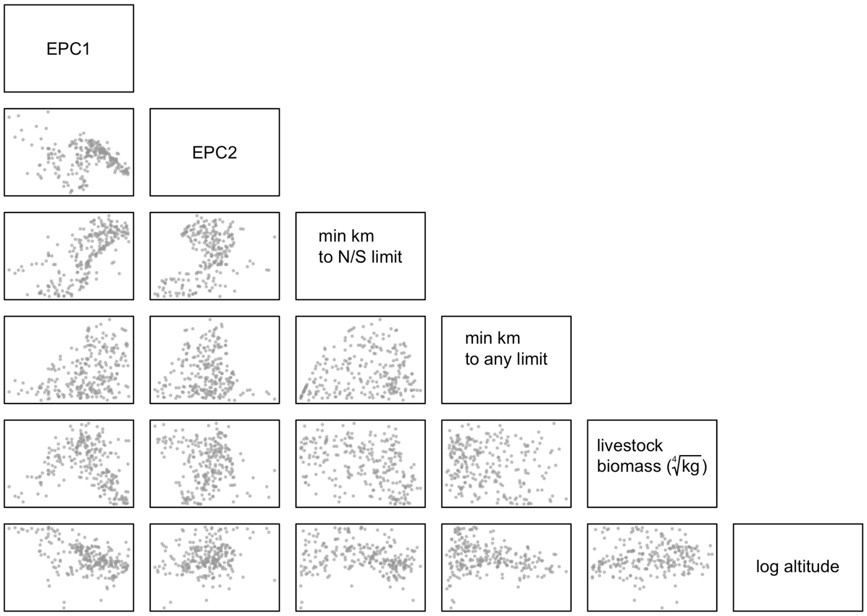

S6. GAM results

Figure S7. Black lines and grey bands show the fitted means and 95% confidence intervals for remaining predictors for the other top GAMs (minimum distance from the northern and southern limit is excluded); data are scaled by sample size and colored by WBC PC1.

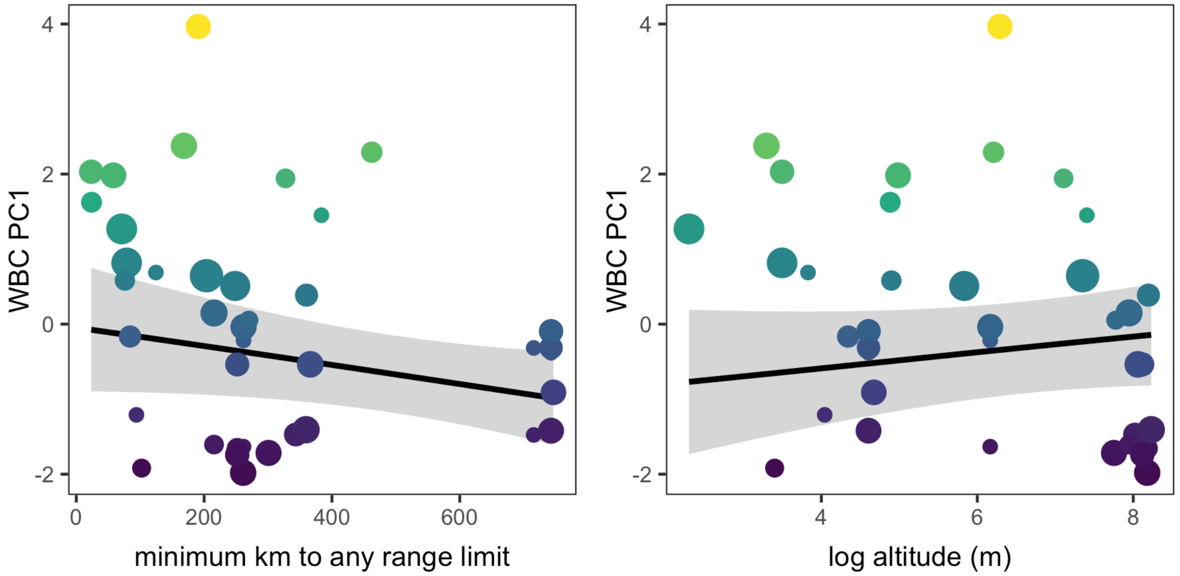

Figure S8. The spatial correlogram shows the estimated values of Moran's *I* for residuals from the top GAMs as a function of inter-site distance. The black line and grey band show the fitted mean and 95% confidence interval using separate GAMs with Moran's *I* as the outcome.

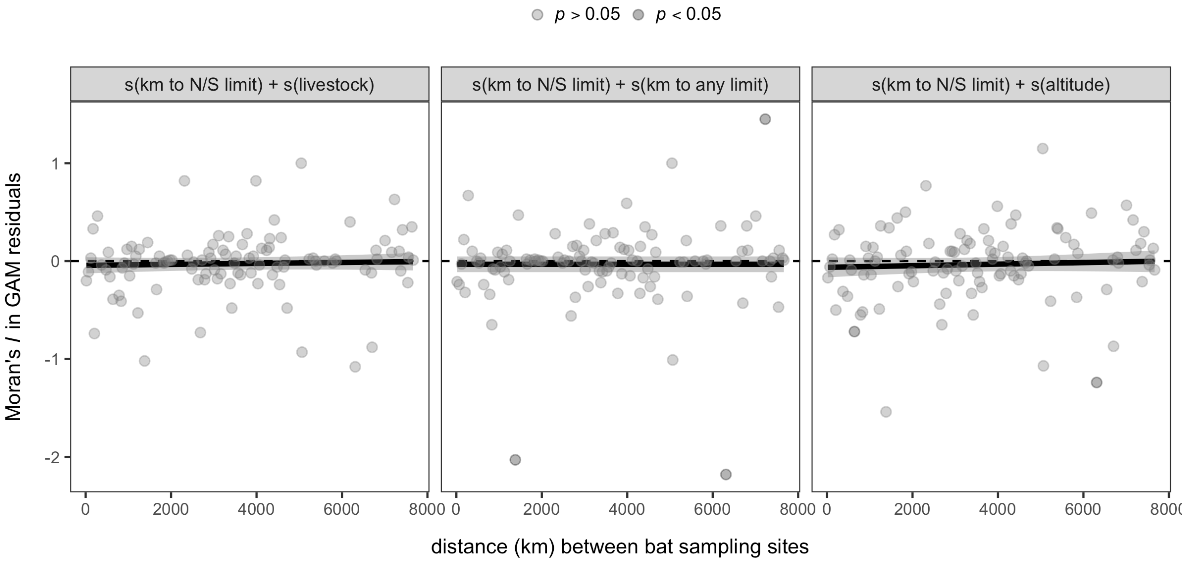

S6. Holding time relationships

Figure S9. Relationships between mean handling time classifications per site and vampire bat leukocyte profiles for our field-collected data (*n*=33). Points are scaled by sample size, and bands show the 95% confidence intervals and predicted mean WBC PC1 from a weighted linear model.

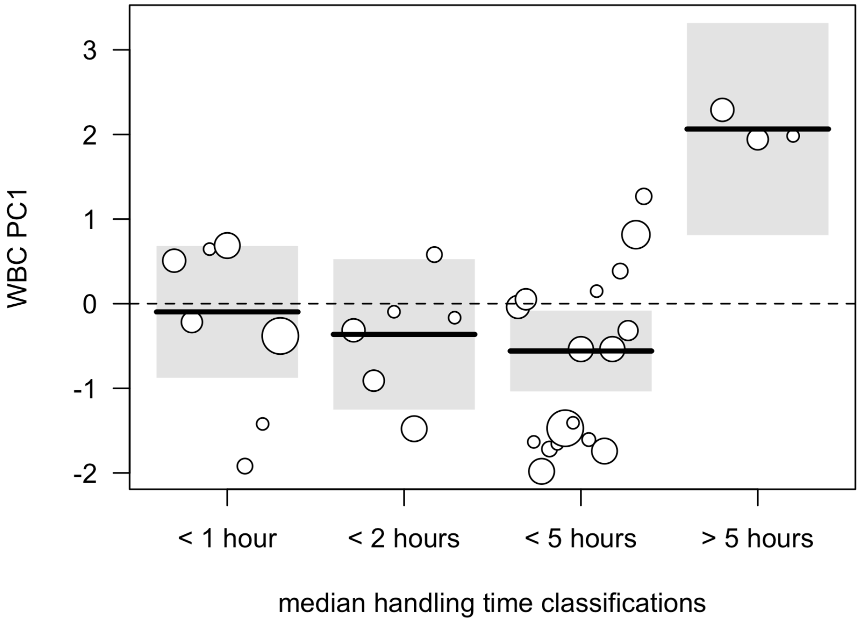

Figure S10. Results from refitting the correlogram and top GAM excluding site data where the median handling time exceeded five hours and literature data (for which handling time was not reported). Point size and color follow the main text.

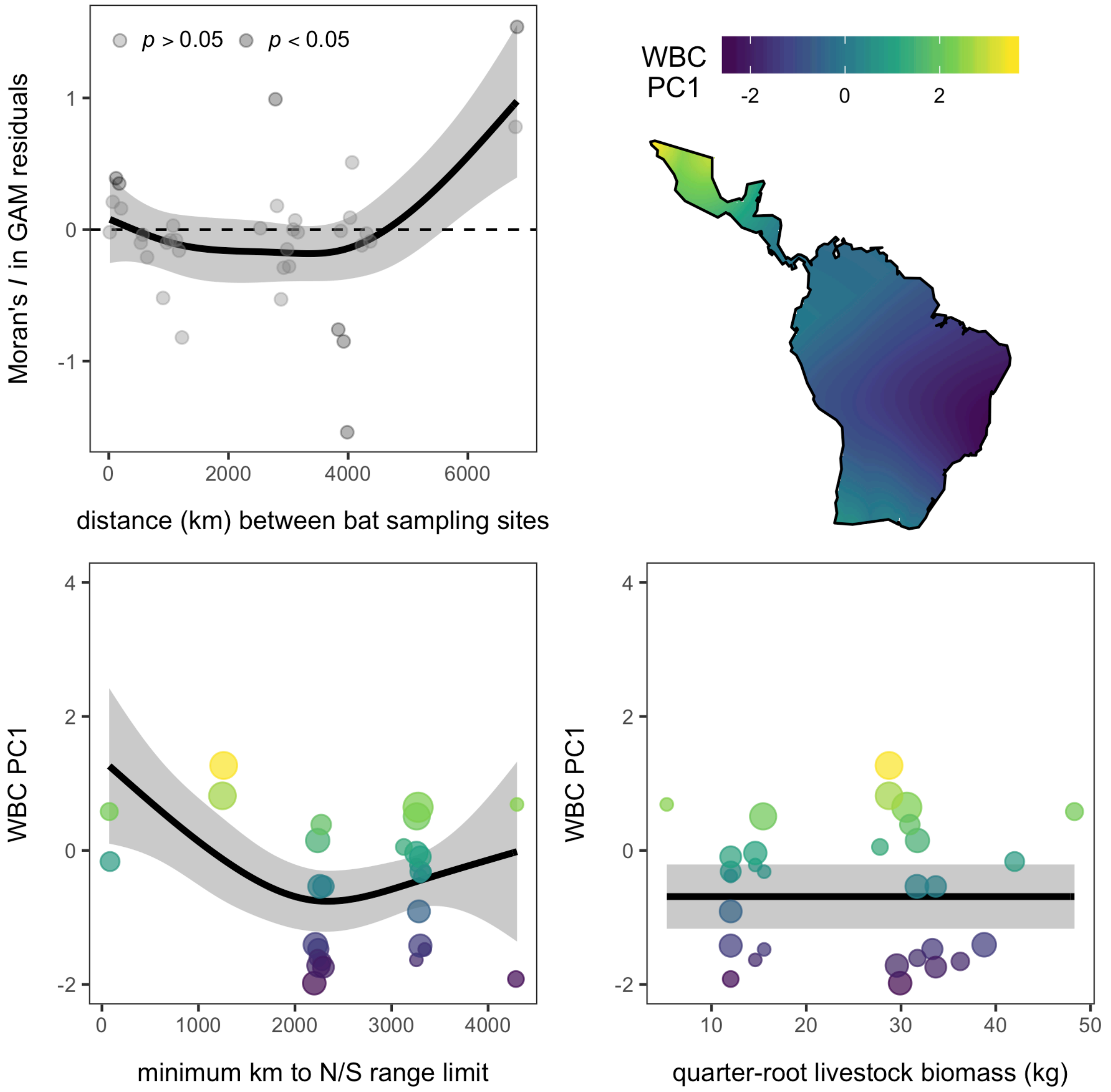

Figure S11. Results from refitting the correlogram and top GAM only excluding site data where the median handling time exceeded five hours (literature data, for which handling time was not reported, are retained). Point size and color follow the main text. We still observed significant variation in Moran's *I* as a function of distance ($F_{3.4,6}$=6.59, p<0.001), with most autocorrelation occurring at large rather than small distances. The effect of latitudinal range limits is numerically similar to that in the main text ($F_{4.6,6}$=9.37, p<0.01), although the effect of livestock biomass is slightly weaker ($F_{0.7,6}$=0.37, p=0.07). The spatial predictions closely match those in Figure 4.

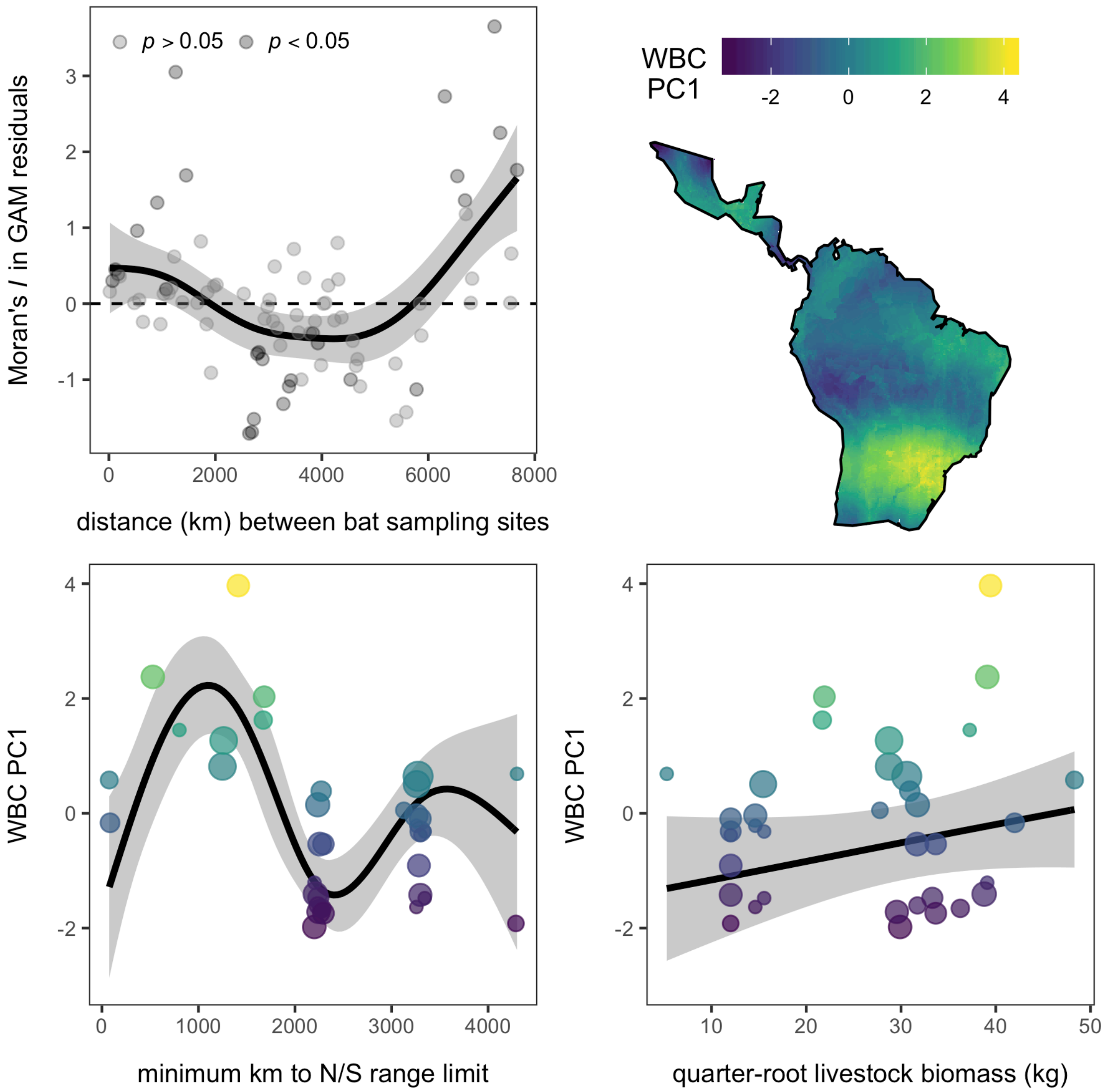

Supplement: icz007_Supplementary_Data [file icz007_supplementary_data.pdf]
